# Virtual Patients in a Behavioral Medicine Massive Open Online Course (MOOC): A Case-Based Analysis of Technical Capacity and User Navigation Pathways

**DOI:** 10.2196/mededu.4394

**Published:** 2015-09-10

**Authors:** Andrzej A Kononowicz, Anne H Berman, Natalia Stathakarou, Cormac McGrath, Tomasz Bartyński, Piotr Nowakowski, Maciej Malawski, Nabil Zary

**Affiliations:** ^1^ Department of Learning, Informatics, Management and Ethics Karolinska Institutet Stockholm Sweden; ^2^ Department of Bioinformatics and Telemedicine Jagiellonian University Medical College Kraków Poland; ^3^ Department of Clinical Neuroscience Center for Psychiatry Research Karolinska Institutet Stockholm Sweden; ^4^ Academic Computer Center Cyfronet AGH Kraków Poland; ^5^ Department of Computer Science AGH University of Science and Technology Kraków Poland; ^6^ Mohammed VI University of Health Sciences Casablanca Morocco

**Keywords:** computer-assisted instruction, education, medical, medical informatics applications

## Abstract

**Background:**

Massive open online courses (MOOCs) have been criticized for focusing on presentation of short video clip lectures and asking theoretical multiple-choice questions. A potential way of vitalizing these educational activities in the health sciences is to introduce virtual patients. Experiences from such extensions in MOOCs have not previously been reported in the literature.

**Objective:**

This study analyzes technical challenges and solutions for offering virtual patients in health-related MOOCs and describes patterns of virtual patient use in one such course. Our aims are to reduce the technical uncertainty related to these extensions, point to aspects that could be optimized for a better learner experience, and raise prospective research questions by describing indicators of virtual patient use on a massive scale.

**Methods:**

The Behavioral Medicine MOOC was offered by Karolinska Institutet, a medical university, on the EdX platform in the autumn of 2014. Course content was enhanced by two virtual patient scenarios presented in the OpenLabyrinth system and hosted on the VPH-Share cloud infrastructure. We analyzed web server and session logs and a participant satisfaction survey. Navigation pathways were summarized using a visual analytics tool developed for the purpose of this study.

**Results:**

The number of course enrollments reached 19,236. At the official closing date, 2317 participants (12.1% of total enrollment) had declared completing the first virtual patient assignment and 1640 (8.5%) participants confirmed completion of the second virtual patient assignment. Peak activity involved 359 user sessions per day. The OpenLabyrinth system, deployed on four virtual servers, coped well with the workload. Participant survey respondents (n=479) regarded the activity as a helpful exercise in the course (83.1%). Technical challenges reported involved poor or restricted access to videos in certain areas of the world and occasional problems with lost sessions. The visual analyses of user pathways display the parts of virtual patient scenarios that elicited less interest and may have been perceived as nonchallenging options. Analyzing the user navigation pathways allowed us to detect indications of both surface and deep approaches to the content material among the MOOC participants.

**Conclusions:**

This study reported on first inclusion of virtual patients in a MOOC. It adds to the body of knowledge by demonstrating how a biomedical cloud provider service can ensure technical capacity and flexible design of a virtual patient platform on a massive scale. The study also presents a new way of analyzing the use of branched virtual patients by visualization of user navigation pathways. Suggestions are offered on improvements to the design of virtual patients in MOOCs.

## Introduction

### Background

The rise of interest in massive open online courses (MOOC) is remarkable. What started as an experiment in connectivist learning theory turned, in just a few years, into a phenomenon involving several million participants and the most prestigious universities [1,2]. Even though the trend has met with criticism [3] and there are already signs of fading enthusiasm [4,5], the changes witnessed in terms of the openness of education to massive participation are unlikely to be reversed.

Health sciences, traditionally slower in adaptation of new trends in education, are inspired by these changes as well [6,7]. A recent systematic review identified nearly 100 health-related MOOCs conducted in 2013 [8]. The motivations of medical universities for participating in such initiatives varies but may involve reaching out to appropriate but less privileged learner groups, possibly to bridge the language gap between patients and their health care providers or to increase the impact and visibility of the university in a particular field [6-8]. MOOCs are also seen as a tool in the “flipped classroom” curriculum reform, which requires students to engage in self-directed online preparations prior to face-to-face learning activities. This frees up time for hands-on training, group work, and individual consultation with teachers on the campus [7,9].

The currently predominant type of massive open online courses, called xMOOCs, has been criticized for building on bite-sized video lecture clips, textbooks, and multiple-choice questions—regarded as a rather outdated form of learning [3]. This development can be partly explained by the decontextualized technical confines offered in generic-purpose MOOC environments. However, there are several discipline-specific information technology tools which could vitalize the learning activities without requiring much attention from the instructors. One of these tools, available for health training, is virtual patients [10].

Virtual patients have shown a positive effect on learning [11,12] and are increasingly used at medical faculties [13,14]. Although definitions vary, virtual patients are most commonly understood as interactive computer simulations of real-life clinical scenarios for medical training, education, or assessment [15]. Several types of virtual patients can be constructed, depending on the applied technology and target competency; these include virtual patient games, high fidelity software simulations, and virtual standardized patients [16,17]. To teach clinical reasoning and decision-making, the most common type involves interactive patient scenarios that employ simple Web-based technologies [17].

We began with a prior theoretical analysis of the idea of embedding virtual patients in MOOCs, from an educational point of view [18] and continued with a discussion of technical mechanisms for the integration [19]. We regarded the extension of platform functionality beyond the standard tools as a major risk considering the sheer numbers of students. Preparation for a MOOC thus requires recruiting a support team to clear pedagogical and technical hurdles [9]. From a different perspective, the great quantity of participants could also offer an opportunity to identify patterns in virtual patient use, which normally remains largely unnoticeable due to the small sample size of students in the traditional classroom. A recent systematic review of literature in the field of MOOCs has concluded that “while there is research into the learner perspective neither the creator/facilitator perspective nor the technological aspects are being widely researched” [20]. We aim to address these needs by reporting on our technical experience in organizing what to our knowledge is the very first health-related MOOC including virtual patients.

### Objectives

The primary objective of this paper is to present an in-depth analysis of the technical preparations required to include virtual patients in a MOOC. This will inform the parameters for future preparatory tests and suggest solutions for dealing with the intensive usage of information technology infrastructure resources while offering a server-side educational component for a massive audience. The second objective is to identify ways of highlighting the different navigation pathways of virtual patient interactive use. We hope that this study will reduce the technical uncertainty related to such extensions, indicate aspects that could be optimized for a better learner experience, and also raise prospective research questions by describing indicators of virtual patient use on a massive scale.

In particular, we are interested in answering the following two research questions: (1) What are the information technology challenges and technical solutions for offering virtual patients in a MOOC? (2) How can user navigation pathways be presented for virtual patients integrated within a MOOC?

## Methods

### Setting

This report is a case study carried out at Karolinska Institutet (KI), a Swedish medical university, in the second half of 2014. KI was the first Scandinavian university to join the edX consortium launched by Massachusetts Institute of Technology and Harvard to create and disseminate MOOCs [21]. Once the agreement with edX was concluded in the summer of 2013, an internal call led to the selection of two courses for the first wave of KI MOOCs. One of them was “KIBEHMEDx: Behavioral Medicine—a Key to Better Health,” presenting the science of changing behavior to improve health and quality of life [22]. This course was selected as a target for introducing virtual patients due to its clinical, case-based character.

Being part of the edX consortium required using the edX MOOC platform to host the course [23]. The platform supports presenting videos, multiple-choice questions, and facilitation of online discussion; however, it has no direct support for presenting interactive patient scenarios. Our prior research has shown that this integration challenge can be resolved by using an IMS LTI-interface [19]. For the virtual patient platform we selected an open-source solution: OpenLabyrinth [24]. This platform is the most advanced, freely available interactive patient scenario system with a long history of use in educational activities and research projects [25-28]. The platform supports the branching paths navigation model, meaning that learners are presented with a clinical case in which they can select from a number of alternative options that lead to individual learning trajectories [29]. Version 3.1 was selected following a recommendation by the developer as the most stable release at the time the course was prepared. The standard graphical layout of the system was altered to fit the edX design using the built-in skin mechanism.

**Figure 1:**
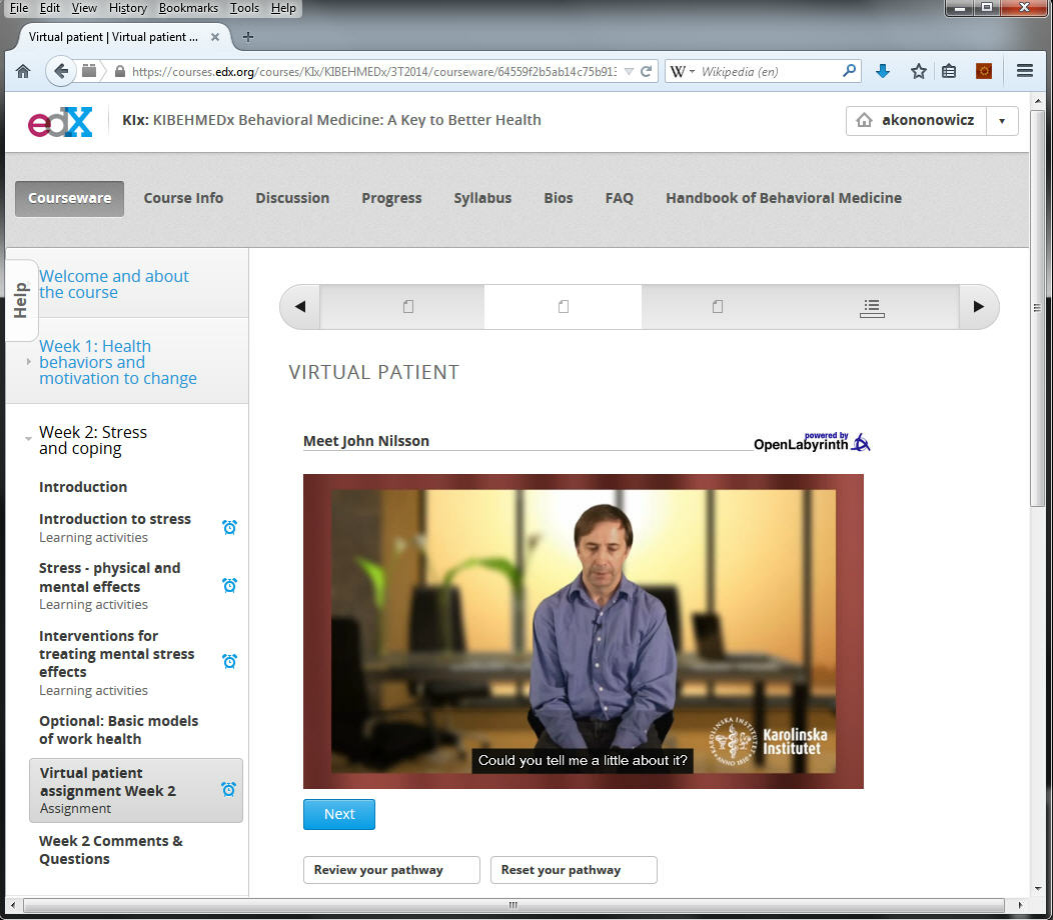
Virtual patient presented in the edX Behavioral Medicine course.

The Behavioral Medicine course was designed for a 5-week run. In order to increase active learning opportunities it was decided that weeks 2 and 3 would be illustrated by virtual patients in the form of interactive patient scenarios. The week 2 scenario dealt with treatment of stress-related symptoms; week 3 with treatment of sleep problems. These scenarios were presented as separate cases but both were connected by the story of John Nilsson, a high school teacher suffering from stress and sleeping problems (Figure 1).

The virtual patient scenarios consisted of 80 and 61 screen cards or nodes, respectively, containing text description, decision elements, free-text assignments, multiple-choice questions, and videos (Table 1). The videos were created for the purpose of this course and involved a professional actor, two clinicians, and a film team. Video length varied from 16 seconds to 6 minutes 39 seconds. All videos were hosted on YouTube and embedded in the virtual patient scenarios using an internal frame. Some of the videos from the first week (week 2 of the course) were repeated in the week 3 scenario, forming review nodes. Decision nodes represent screen cards that allow the user to select how to proceed, based on at least two options. The branched navigational structure of virtual patients was designed in the VUE (v3.2.2) editor [30] and then exported to OpenLabyrinth. The possibility of following the same branching option twice was blocked to prevent cycles. The virtual patient activity for each of the two course weeks was planned for approximately one hour. Students were asked to self-report, following the edX honor code, by indicating that they spent at least 30 minutes per week interacting with the virtual patient.

The Behavioral Medicine MOOC started on September 9, 2014, and lasted 5 weeks, until October 14. The week 2 virtual patient was available for the first time on September 16 and the week 3 virtual patient became available on September 23. All services were active for two more weeks (until October 28) for a tapering period.

### Technical Infrastructure

The OpenLabyrinth system is successfully used at several medical universities worldwide. However, to the best of our knowledge, it has not been used as part of a large course yet. As a recent systematic review has shown, an average MOOC involves 43,000 enrollments, and there have been reports of courses with over 200,000 registered users [4]. The typical dropout rate is very high with just 6.5% of participants finishing the course on average [4]. It is also known that user activity is not evenly distributed, with large peaks before deadlines for graded homework [2]. Since MOOCs are often characterized by demands for computational power in bursts, we decided to use a cloud infrastructure as a solution that matches the expected requirements well.

Cloud computing is a way of providing computing resources as services that can be created on demand, usually in the form of virtual servers (machines). Clouds have been applied by industry to dynamically scale Web applications to respond to varying peaks in workloads. They have also been successfully used in scientific and healthcare applications, where they allow for obtaining computing resources quickly to compute intensive simulations or data analysis tasks [31,32]. In medical research, a good example is the Virtual Physiological Human (VPH)-Share project, which offers a cloud platform for hosting and sharing computational models of the VPH research community, providing on-demand access to computing services [33,34].

To evaluate the performance and scalability of OpenLabyrinth, we used Gatling (v1.5.5) [35], to carry out stress tests of the system on 8CPU/16GB RAM server using a simple virtual patient test case consisting of 10 nodes and content similar to that expected in the final version of the case. We simulated 50, 100, 150, and 300 users traversing three random paths, changing the nodes with one request per second on average. In the 300-user test, this resulted in 14 requests per second at the highest peak during the stress test time lasting 17 minutes. The number of 300 concurrent users was regarded as sufficient for the maximal load since a previous report from the 6.002x course, which had an enrollment rate of 154,000, indicated that the reported peak of activity per day was 5000 unique certificate earners; divided by 24 time zones this generated an estimate of slightly above 200 active users an hour [2]. To reflect the nonuniform distribution of users in time zones we increased the upper boundary to 300 users.

The stress tests showed a very good response time in all cases (around 100 ms), low RAM (max 460MB) and processor use (max 1.3CPU) but around one percent (0.66%) of requests for 300 concurrent users led to a 404-page error. This was traced to a database deadlock problem. The error rate for 50 users was significantly lower (0.02%). Since it was not feasible for us to find the source of the deadlocks in the given timeframe, we decided to mitigate the risk of this error by reducing the number of concurrent users using cloud technologies. The idea was to have more numerous but less powerful virtual servers to share the user requests in a balanced way and thus decrease the likelihood of database problems.

**Figure 2:**
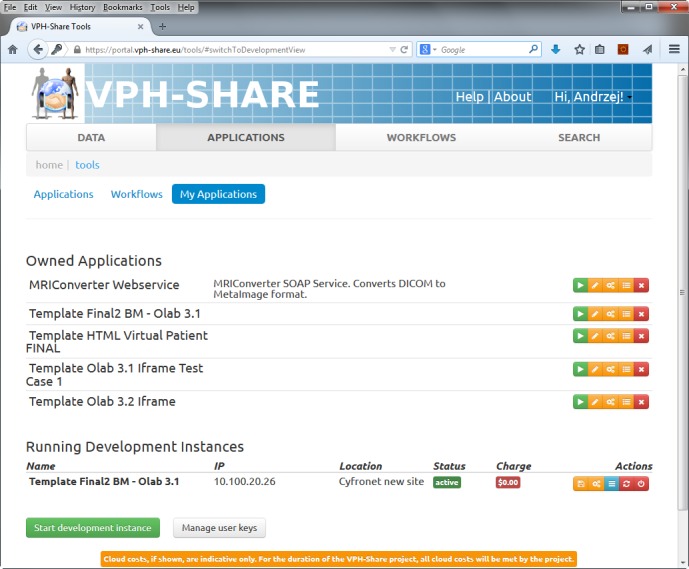
VPH Share cloud platform with virtual patient system templates prepared for the Behavioral Medicine MOOC and a running instance of one of the templates on a virtual server.

**Figure 3:**
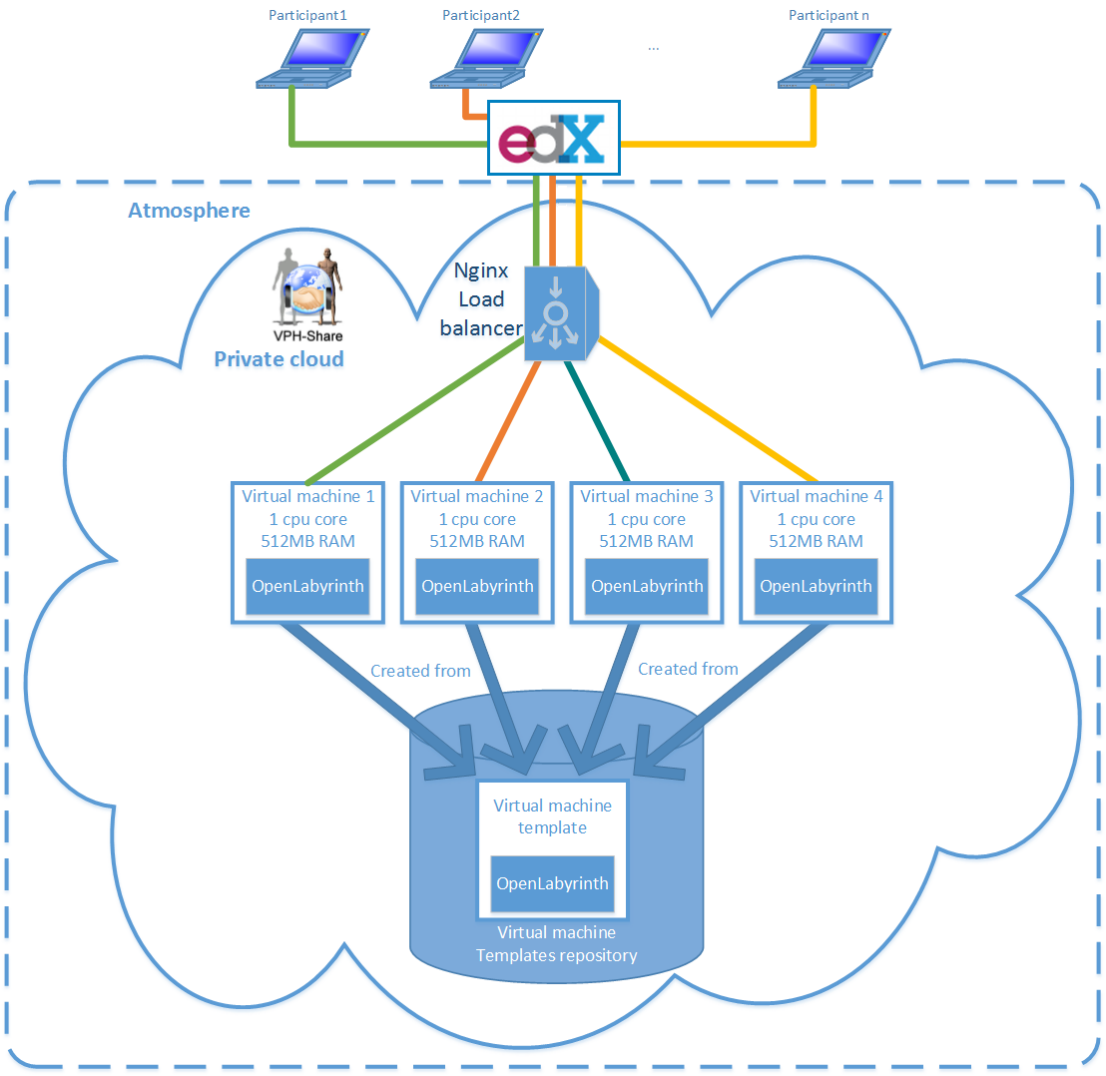
Cloud architecture for the Behavioral Medicine MOOC.

For the implementation we approached the VPH-Share project, specialized in offering cloud services for biomedical applications [34]. Their cloud management solution—Atmosphere [36]—enables flexible design of virtual server templates (images) and their execution as atomic services in a number of software and hardware configurations (CPU and RAM) [37]. We developed a virtual server template based on the Ubuntu (v13.10) Linux distribution (Apache v2.4.6; MySql v5.5.37; PHP v5.5.3) and with OpenLabyrinth (v3.1) preinstalled (Figure 2). A load balancer (nginx) was instantiated to evenly distribute user requests between template instances. For the start-up we decided to use four micro instances of virtual servers (1CPU; 512MB RAM) in parallel with the possibility of increasing this number in case of a higher than expected workload. The cloud infrastructure was hosted by Academic Computer Centre Cyfronet AGH in Kraków (Poland), running OpenStack cloud software [38] (Figure 3). The use of the VPH-Share platform should not be considered a limiting factor as it has many similarities in functionality with commercial providers like Amazon EC2, and the virtual server templates are in fact fully transferable between the solutions.

As an emergency backup solution in case of unexpected technical problems, we had prepared an alternative virtual server template consisting merely of the Web server with a preinstalled HTML version of the virtual patients, not requiring the database system. This version was designed by writing a script exporting OpenLabyrinth cases to a set of static HTML web pages. Obviously, this version had fewer computational requirements; however, it also had limitations in terms of restricted functionality, as tracing user sessions or recording students’ answers was not possible. In the end it turned out to be unnecessary to use this template during the MOOC, but it provided security for the project team.

### Data Collection and Analysis

Web server and database logs as well as user sessions were recorded in the OpenLabyrinth system. The data were archived and aggregated from the four virtual servers after the tapering period had ended. There was no need to add new parallel servers. The Web server logs were parsed using a simple Java language script and postprocessed in Excel (Microsoft). Spearman correlation coefficients were calculated in Statistica v10 (2010) (Statsoft), using a significance level of 5%.

User pathways were analyzed by an on-purpose project-designed analytic tool developed in Java by one of the authors of this article (AAK). The developed tool visualizes the results by displaying them as numerical and grayscale values in the navigation graph. The structure of the graph is read from VUE files used previously in designing the virtual patients. Next, the XML content from VUE files is copied and modified based on the user session statistics from OpenLabyrinth and once again opened in VUE to display the result. Four different types of visual analyses were generated: (1) number of visitors in each screen card of the virtual patient, (2) pathway exit points: percentage of visitors who ended the session in the given node, (3) time in seconds spent in each screen card on average, (4) percentage of visits selecting individual decision options and branching ability of decision nodes.

In order to evaluate participant satisfaction with the course in general as well as the virtual patient experience and to confirm the quality of technical capacity offered in the MOOC, the participants were surveyed for their opinions on using virtual patients via an anonymous questionnaire to which invitations were distributed immediately after the course. The Likert-scale questions were analyzed using Excel for descriptive statistics. Free-text comments detailing the technical issues encountered by course participants were analyzed qualitatively for recurring themes.

Data on user enrollment and declared completion were acquired from the edX platform statistics (edX insights). In this study, we did not trace the link between sessions and user demographics or learning outcomes and treated user data entirely anonymously. This type of research does not require explicit permission of an ethical review board according to Swedish law (Act 2003:460).

## Results

### General Statistics

The number of enrollments reached 19,236 but just 4586 (23.84%) logged in during the first week of the course. On the official closing date of the course (October 14), 2317 (12.05% of the total enrollment rate) and 1640 (8.53%) participants had declared their respective completion of the week 2 and week 3 virtual patients. This number kept growing after the course was officially closed in the tapering period. The honor code certificate for the whole course was earned by 740 participants, or 3.85% of the total number of original enrollments. The most frequent participant countries of residence were: United States (27.61%), India (8.97%), and United Kingdom (4.84%). See Multimedia Appendix 1 for a geographic breakdown of participation.

### Server Load

**Figure 4:**
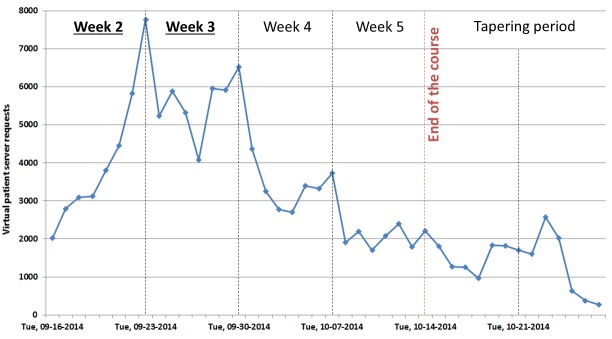
Virtual patient server requests per day in the Behavioral Medicine MOOC.

Figure 4 presents the number of server requests per day in the course lifetime and tapering period. As planned, the virtual patient service started in the second week of the course. The focus of the course instructors on virtual patients was in week 2 and 3. The peak of activity in the virtual patient system was on September 23—the release date for the second virtual patient scenario—with 7768 page requests per day corresponding to 359 unique user sessions. During that day in the most active hour (17:00-18:00 CET) the service had 875 page requests (24 unique user sessions).

**Figure 5:**
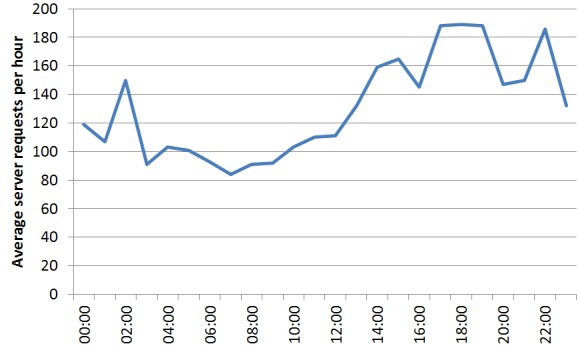
Average virtual patient server requests per hour in the Behavioral Medicine MOOC.

In general, the most active hour of the day was 18:00-18:59 CET with 181 server requests on average. The least active time was 07:00-07:59 CET with 84 server requests on average (Figure 5).

We also analyzed the number of errors reported in the Web server logs. Out of the 131,303 server requests, only 35 could be traced as having been caused by database problems (0.03%).

### User Pathways

**Figure 6:**
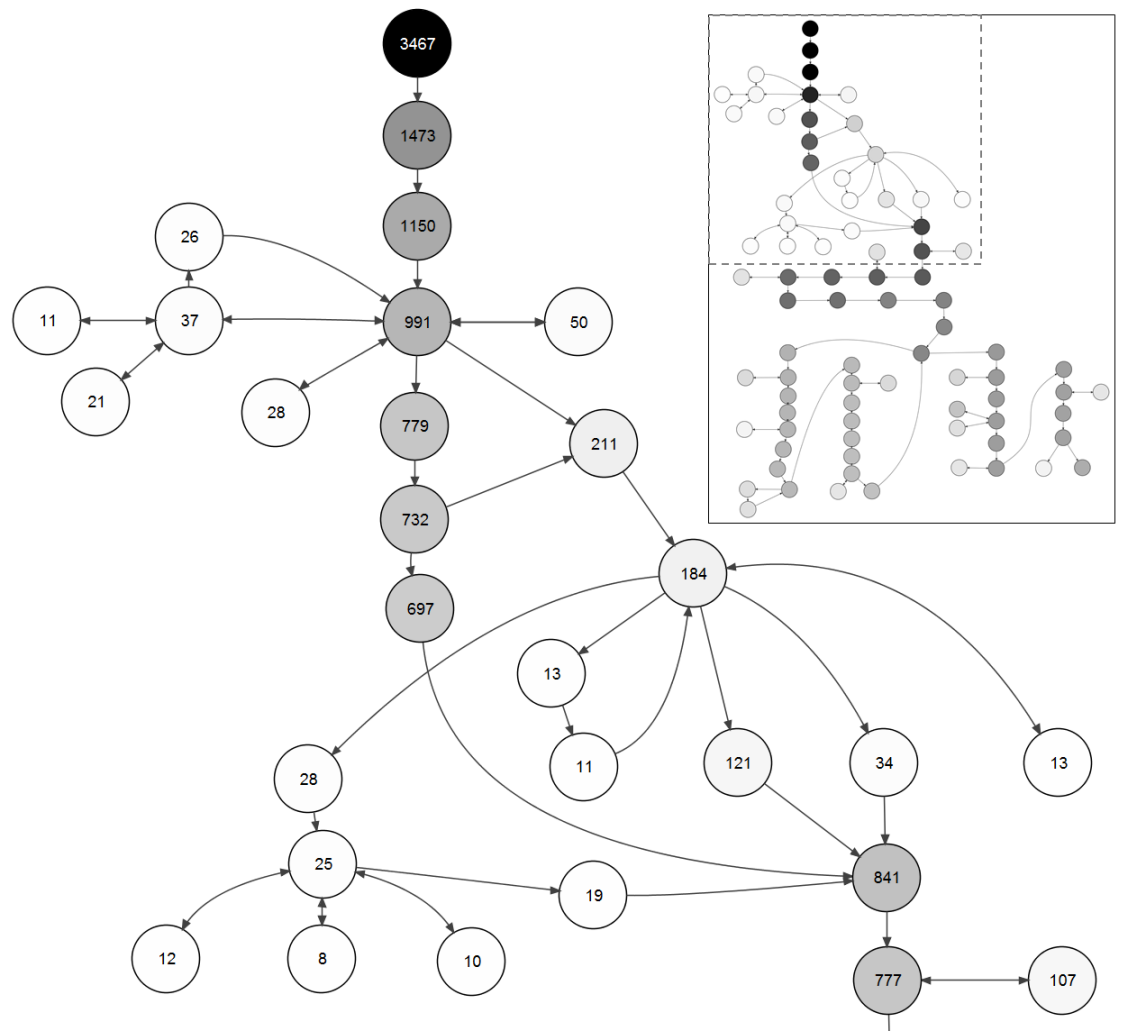
Number of visitors in screen cards of week 2 virtual patient.

The analysis of the number of visitors to the virtual patient scenarios shows a relatively high number of initiated sessions: 3467 in week 2 and 2201 in week 3 (Figure 6). At the same time the bounce rate (number of users leaving just after opening the start page of virtual patients) is also high: 1994 out of 3467 (57.51%) in week 2 and 1486 out of 2201 (67.51%) in week 3. Around 75% of sessions ended before visiting 10% of the total number of screen cards in both virtual patients. The most popular pathway (and shortest at the same time) through the week 2 scenario is visible by the most intensively shaded line of nodes in the visualization in Figure 6. This pathway was designed by the case authors as containing the best options from a clinical point of view, based on course content. It consists of 30 screen cards. Four hundred twenty participants (n=420) reached the final nodes of the case. In week 3, the shortest and best path was also the most popular one. Three hundred forty-five (n=345) participants reached the final nodes of the week 3 case.

The frequency distribution of the number of visited screen cards in one session had a local peak around the length of the critical path (number of screen cards in the shortest pathway connecting the start and end nodes). The frequency distribution for week 2 had an additional peak around 51 nodes, corresponding to the group of students who selected a branch with 16 nodes for additional explanations. The frequency distribution of user pathways longer than the length of the critical path drops steadily, reaching the zero level at 79% (week 2) and 90% (week 3) of the total number of nodes in the scenarios.

Table 1 shows the time spent by users on average in different screen card types. Participants spent the most time (approximately 2 minutes) on screen cards requiring entry of a free text answer to a question. This occurred despite the fact that each free text question displayed a clarification stating that no individualized feedback would be provided in response to student entries. Interestingly, we observed a moderately strong correlation between the average percentage of viewed video time and shortest distance of the screen card to the start node (R=0.55; *P*<.001). The average time spent on video nodes was shorter than the actual length of the video when the node was closer to the start than the end of the interaction with the virtual patient but was longer than the actual length for videos closer to the end node.

**Table 1:** Average time spent on screen card types.

	Week 2		Week 3	
Screen card type	Count	Time [s]	Count	Time [s]
Decision node	19	28.6	11	27.2
Free text question	6	120.3	5	131.6
Multiple choice question	1	66.4	2	23.1
Review	0	-	4	124.3
Text	35	17.5	25	20.4
Video	19	98.8	14	73.4

In the week 2 scenario, the shortest possible path from the start to the end node required 37 minutes, but the average session in which the case was completed (containing one of the end nodes) lasted 48 minutes. One hundred twenty-one (n=121) sessions that completed the virtual patient were longer than one hour. For the week 3 scenario, the shortest path consisted of nodes taking 27 minutes on average, where the mean case completion session was 39 minutes long, with 56 sessions longer than one hour.

**Figure 7:**
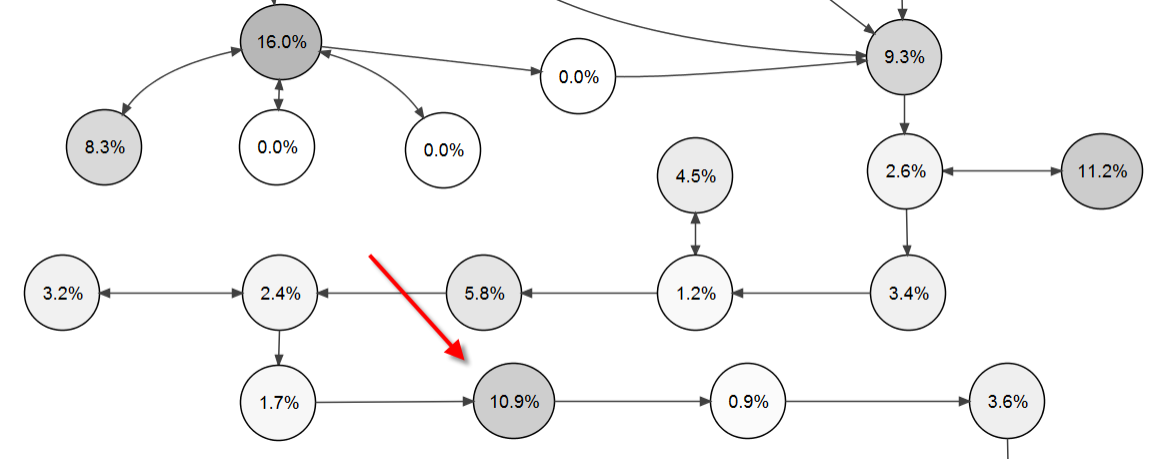
Percentage of visitors who left the virtual patient after visiting the particular screen card.

Figure 7 shows visualization of exit points in the week 2 virtual patient, displaying the percentage of visitors who left the virtual patient after visiting the given screen card. The percentage is relative to the total number of users entering the node. We have highlighted a node with a high dropout rate, possibly due to a challenging task in this screen card. A review of the specific node indicated that it contained a time-consuming free text question, referring to previous videos, which might have discouraged the participants from continuing their work with the virtual patient scenario.

**Figure 8:**
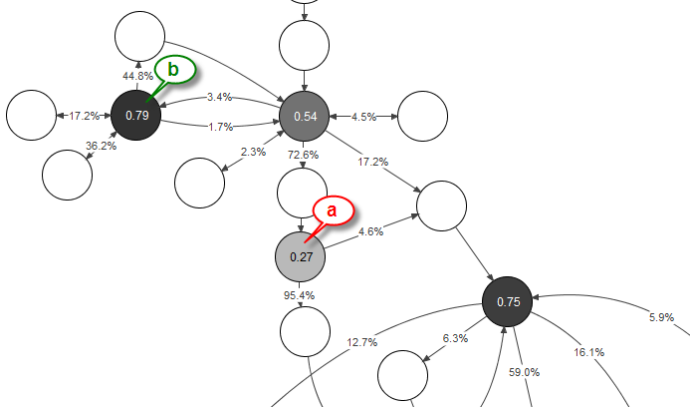
Visualization of learner choices in decision nodes.

Figure 8 shows a visualization of choices made by the course participants in the week 2 virtual patient. The links (edges) leaving decision nodes are indexed by the percentage of visits following this option relative to the total number of outgoing connections. The decision node is labeled by a heuristic value calculated as information entropy from the percentage of selected options, divided by the maximal information entropy for the given number of branches. We use this value as a benchmark for branching quality as it has its maximum value 1 for perfectly even distributed user selections. The node highlighted by *a* in Figure 8 is an example of poor branching as the alternative option is selected in just 4.6% of visits which may indicate a nonchallenging choice. Correspondingly, the branching value is low (0.27). In contrast the node highlighted by *b* in Figure 8 has a more evenly distributed user count, indicating interesting and not obvious choices (heuristic value 0.79).

### Survey Results

We received 479 responses to the participant survey (2.49% of all enrolled and 20.67% of those who declared they had completed at least one virtual patient). Multimedia Appendix 2 contains a detailed summary of the responses. For the great majority, using a virtual patient was a new experience (87.3% declared they had not used virtual patients before and 11.5% had used virtual patients prior to the course). A great majority strongly agreed (58.5%) or agreed (24.6%) with the statement that the virtual patient was a helpful exercise in the course. Only 1% of participants disagreed or strongly disagreed. The level of difficulty of the virtual patients was just right for the majority of respondents (neither too easy nor too difficult: 62.8%). It was difficult to very difficult for 21.1% and easy to very easy for 12.1%.

Eighty-one percent (389 out of 479; 81.2%) of surveyed participants did not encounter any technical problems while using virtual patients. Nevertheless, it was surprising that a relatively high percentage (77 out of 479; 16.1%) reported experiencing technical issues. The dominating themes in the free text description of technical issues were (1) problems with videos, (2) Internet connection problems, (3) problems with broken sessions, and (4) unrelated problems. Each of these themes contained around 15 comments. The video problems related to the inability to access YouTube videos in some countries or the inability to download them offline. Sometimes the videos did not seem to start properly, got stuck, or played only the audio track—these problems could be explained by slow Internet connections or compatibility issues with Web browsers on some platforms. The problems explicitly classified as Internet issues referred in general to the inaccessibility of broadband connections in regions of the world such as India or rural Canada. The issues classified as broken sessions were most likely related to the virtual patient platform and manifested by nonsaved user answers, page display errors, unexpected session reset, or (in two reported cases) surprising redirection between nodes. Some of the problems could be explained by session timeout or browser issues but they could just as likely be due to previously identified software problems in the platform. Luckily, these difficulties were not reported often and did not influence the general positive picture of the learning experience. Lastly, the technical complaints also contained issues not directly related to the technical side of virtual patient scenarios, like language issues, difficulties in posting comments on the discussion board, or complaints about videos that were not part of the virtual patient scenarios.

## Discussion

### Principal Findings

This case study presents experiences collected while organizing what is probably the first MOOC including virtual patients. The scope of the report is on virtual patient usage expressed in terms of required technical capacity and user navigation pathways to inform preparations of further, similar courses. The general impression after introducing virtual patient scenarios into the Behavioral Medicine MOOC was positive. The great majority of participants regarded virtual patients as a helpful exercise in the course, and the declared completion rate of the virtual patients activity (12.05% and 8.53% out of 19,236, weeks 1 and 2, respectively) was relatively high in relation to the total course completion rate (3.85%). We received many free text comments in the discussion forums praising this learning resource (Berman et al, manuscript in preparation). The course organizers will retain this activity for continued runs of this MOOC and have begun using the virtual patients in their traditional campus-based teaching activities.

From the organizers’ perspective, the intimidating number of participants at start-up was not as challenging from the technical perspective as expected. One contributing factor is the high dropout rate typical for MOOCs, and the more evenly distributed frequency of requests than expected due to the truly global reach of the learning activity. In the end, the technical infrastructure was loaded, in terms of concurrent users, 20 times less intensively than anticipated as the worst-case scenario. The server capacities of one virtual machine would be most likely have been sufficient to host the course even when considering the database problems noted in the course live run.

Considering the virtual patient statistics obtained, the necessity of using cloud infrastructure might be questioned. However, several benefits to this solution were confirmed in this study. A platform-as-service saves costs, as it was not necessary to purchase any new hardware for the MOOC to guarantee a dedicated server for the virtual patient platform. The possibility of scaling the solution—horizontally (number of CPU and RAM) and vertically (number of instances)—provided us with security in case of higher than expected popularity of the course. The openness of MOOCs suggests the real need of taking such precautions. The solution of preparing different types of virtual server templates was very helpful. This provided flexibility for the course organizers in terms of switching between different services outside the MOOC platform, depending on how the course evolves in real time. It also saved time while reusing the services for other occasions (eg, for on-campus classes with small groups of students). The idea of creating micro-instances of virtual servers to circumvent problems with concurrent access to legacy code is new and has not been discussed in the literature before [39]. The presented integration is likely to be succeeded by more computationally demanding integrations of simulation in virtual patients [40]. For such scenarios the use of cloud services will be indispensable.

The OpenLabyrinth system proved its usefulness in this study as a virtual patient player for a MOOC. The technical problems reported by some students did not seem to overshadow the general positive picture of the activity. The problems we traced as directly related to the OpenLabyrinth environment were not numerous. This would be interpreted differently in the case of commercial courses or high-stake examinations organized on a massive scale. The most often reported problems with low Internet connection for videos or banned YouTube services are not related to the virtual patient software. The course participants were informed by the organizers in the general course rules about restrictions in use of the YouTube service in some countries and offered manual downloads of the video clips outside OpenLabyrinth; however, this solution was not offered explicitly in OpenLabyrinth. This seemed to negatively influence users and suggests other options should be considered for hosting videos. We recommend uploading all videos as separate files to the MOOC platform to be downloaded on demand by participants from countries with blocked access to services such as YouTube. An alternative solution would be to host a video streaming service on a generic cloud infrastructure, but this would considerably raise the technical requirements. We were not able to spot any irregularities which would indicate users of specific web browsers had particular problems using virtual patients in OpenLabyrinth. It is to be acknowledged that we did not optimize the display for mobile devices (tablets, smartphones), a factor which could be an issue for some of the participants. We recommend considering this group in particular in upcoming MOOCs introducing virtual patients.

The course was slightly less frequently visited and completed than an average MOOC. This might be explained by the specificity of the topic and the general trend of larger supply and shrinking interest in MOOCs [4]. The high bouncing rate of virtual patients is not surprising as it includes those who just explored the course’s content without intending to interact with the cases. Among those interested enough to move to the second node of virtual patient, 30% to 50% completed the exercise, which took 45 minutes on average. We interpret this as an objective sign of interest in the task.

One worrisome aspect is the discrepancy between the self-reported completion rate of the exercise and the actual session logs. It was not necessary to complete the virtual patient exercise in order to tick off the assignment; users merely had to spend 30 minutes in the scenario and comment on this on the discussion board. The declared completion rate of the week 2 assignment was 2317, but the number of sessions containing the second node of virtual patient was 1473. This raises doubts regarding how seriously participant declarations are to be taken. The observation of digital dishonesty is not new [41]. It is unclear, however, why participants would act dishonestly when no formal recognition is attached to acquiring an honor code certificate. Future studies should examine this kind of behavior in more detail.

One innovative aspect of this study is the use of visual analytics methods to report user activities in virtual patients. Visual analytics is an emerging trend employing human cognitive abilities to recognise visual patterns in analytical tasks [42]. Visual methods have been used in activity dashboards of learning management systems and in observing patterns of interaction on discussion boards [43]. However, the potential of visual analytics in virtual patients has been so far largely unexplored. The visualizations presented in this paper show a map of the virtual patient scenarios with overlaid navigation pathways, thus including several topological dependencies that would not have been easily noticed in traditional visitor statistics presented in tabular form. This function of depicting the flow of virtual patient activity is highly instrumental for quality control of online education. The coincidence of the most popular pathway with the correct one can be interpreted as a sign of a successful learning process. At the same time it has to be remembered that this indication is biased by the limited number of possible pathways through the case. It has also been reported that some students learn by exploring the wrong options on purpose which might further blur the picture [44]. The detected nodes with high dropout or low branching levels led to discussions about possible changes in the virtual patient structure for future editions of the course.

The observation of the number of visited nodes in user pathways and the moderately strong positive correlation of the percentage of viewed videos to the distance from the start node might suggest the existence of two groups of MOOC participants using virtual patients: learners with either surface or deep approaches to the content material. The existence of such groups is predicted by educational theories [45] and has been observed in e-learning environments [46]. The group we identify as possible surface learners in our MOOC leaves the virtual patient soon after opening the cases (as when 75% of sessions end before 10% of the content is viewed) and do not follow through on the assignments completely (as those leaving the video nodes before the video playback time in initial parts of navigation pathways). The more highly motivated group, identified as about 400 probable deep learners, is visible by the peak in frequency distribution of navigation pathway length with approximate equivalence to the length of critical pathway through the cases. An additional indication of deep learning might be the observed higher percentage of carefully carried out exercises closer to the end nodes (as visible on the example of the average time spent on video nodes). The high motivation of a subgroup of users might also be suspected from the time spent on entering answers to the free text questions (high average time for this type of node) even though there was no hope for individualized feedback or credit for such exercises.

### Limitations

This study has its limitations. There were just two virtual patient scenarios introduced to the course using OpenLabyrinth. The branching potential of virtual patients has not been used to its full extent because of limited resources for developing alternative versions of videos, fixed recommendations of professional behavior which should not encourage too much experimentation, and the novelty of this form of teaching to the subject matter experts of the course. It was also assumed from the beginning that this study would not link session data to user details. The study evaluates the idea of introducing virtual patients in a MOOC at the first level (participation) of Kirkpatrick’s hierarchy [47,48]. We did not evaluate the effects of learning in the MOOC in terms of acquired knowledge, but following the positive outcomes at the first level we plan to conduct evaluations at higher levels and encourage others to do so. As we received 479 responses to the participant surveys (which is 2.49% of the total number of enrolled users), it might pose a high risk for nonresponse bias effect. However, when considering that the evaluation survey was announced as the last step of the whole course and 740 participants earned the certificate, the same number might indicate a high response rate (64.7%) among those who completed the course. The heuristic selected for measuring the branching quality was selected arbitrarily and will be improved in the future, taking into account the baseline level of learner expertise. The time spent by students in individual nodes of the case on actual learning is very difficult to control and should be treated as a rough indicator of the thoroughness of a virtual patient session. Some of the students might have used it for other, unrelated activities. This problem is often encountered while evaluating virtual patients activities [49]. However, as there were no direct incentives for spending more time on particular nodes (the credit was based on an honor code declaration), we assume that this bias is evenly distributed. The impact of any future changes made to improve the quality of virtual patients based on the clues taken from the visual analytics tool will be the subject of studies on future editions of the MOOC.

Future research should focus on observing how participants choose navigation pathways depending on participant-related factors. This could give insight into how experts, expert students, and lay people approach problem solving in a MOOC. It would be interesting to look for correlations between such indicators as branching level and student satisfaction from learning. Technical solutions used in this paper, such as building different variants of virtual patient system templates or using visual analytics methods to improve virtual patient quality are innovative and not yet optimized nor standardized. These could form integral elements of a cloud-based platform dedicated to organizing health-related MOOCs. The introduction of virtual patients to massive audiences, thanks to the large number of participants, opens up new, previously inaccessible venues for experimentation with this learning design with potential for future research on the topic.

### Conclusions

This study reported on the probable first introduction of virtual patients to a MOOC. It positively verified the feasibility of using OpenLabyrinth, an open-source, freely available virtual patient system for a large audience setting. The system can now be added with greater confidence to future health care MOOCs. This report delivered concrete technical parameters (like the number of users per hour) to inform preparatory stress tests carried out prior to extension of health care MOOCs by adding nonstandard server-based interactive components. It demonstrated to the medical community how a cloud infrastructure (using the example of VPH-Share, but generalizable to comparable commercial solutions) can be employed in teaching activities on a large scale to deal with problems with errors in legacy code preventing high numbers of concurrent users, limitations in availability of hardware resources, or the need to prepare and store different configurations of the software tool. The paper further recommends adding to the existing branched virtual patient systems additional components graphically visualizing user pathways by demonstrating the spatial relationship between statistics pertaining to particular nodes. This is an added value to the existing tabular forms enabling the presentation of session statistics in learners’ areas of interest. Nodes rarely visited or visited for a shorter time than expected based on content, as well as branches seldom taken, can be analyzed in a context of preceding and following nodes and in a general overview of the whole case structure. This can be done to increase the attractiveness of the case or detect problems in understanding the content.

The innovation was warmly welcomed by the majority of course participants responding to the survey. A few challenges remained regarding accessibility and slow transfer of YouTube videos and occasional unexpected technical behavior on the part of the virtual patient system. We hope that this article will contribute to the expansion of future health-related MOOCs with interactive elements such as virtual patients.

## Appendix

Multimedia Appendix 1 Geographic breakdown of participation.

Multimedia Appendix 2 Detailed results of the user survey.

## Abbreviations

KI: Karolinska Institutet
MOOC: massive open online course
VPH: Virtual Physiological Human
