# Supplementary material for: Virtual Patients in a Behavioral Medicine Massive Open Online Course (MOOC): A Case-Based Analysis of Technical Capacity and User Navigation Pathways
Source: JMIR Med Educ. 2015 Sep 10;1(2):e8. doi: 10.2196/mededu.4394 (PMC5041343; doi:10.2196/mededu.4394)
Supplement: Multimedia Appendix 1 [file mededu_v1i2e8_app1.pdf]

Appendix 1

Geographic breakdown of participation. Total enrollment 19,236. Information available 18,794.

182 Countries Represented:

United States	5,189	27.61%
India	1,685	8.97%
United Kingdom	909	4.84%
Canada	819	4.36%
Australia	521	2.77%
Germany	422	2.25%
Egypt	392	2.09%
Brazil	354	1.88%
Sweden	348	1.85%
Spain	336	1.79%
China	288	1.53%
Mexico	272	1.45%
Greece	263	1.40%
Russia	256	1.36%
France	249	1.32%
Pakistan	223	1.19%

Philippines	222	1.18%
Netherlands	200	1.06%
Nigeria	180	<1%
Poland	168	<1%
Italy	165	<1%
Hong Kong	159	<1%
Saudi Arabia	159	<1%
Indonesia	158	<1%
Singapore	153	<1%
Japan	144	<1%
Romania	144	<1%
Colombia	142	<1%
Portugal	138	<1%
