# Supplementary material for: Virtual Patients in a Behavioral Medicine Massive Open Online Course (MOOC): A Case-Based Analysis of Technical Capacity and User Navigation Pathways
Source: JMIR Med Educ. 2015 Sep 10;1(2):e8. doi: 10.2196/mededu.4394 (PMC5041343; doi:10.2196/mededu.4394)
Supplement: Multimedia Appendix 2 [file mededu_v1i2e8_app2.pdf]

Appendix 2

Detailed results of the user survey (N = 479)

1. Have you used virtual patients before this course (as teacher or learner) ? If yes, what was your previous experience with virtual patients?

Yes	55	11.5%
No	418	87.3%
[Blank]	6	1.3%

2. Would you agree that virtual patients were a helpful exercise in the course? If yes, in what aspect? If not, please explain.

Strongly agree	280	58.5%
Agree	118	24.6%
Neither agree nor disagree	39	8.1%
Disagree	4	0.8%
Strongly disagree	1	0.2%
[Blank]	37	7.7%

3. Have you experienced any technical difficulties while working with virtual patients in the course? If yes, what were they?

Yes	77	16.1%
No	389	81.2%
[Blank]	13	2.7%

4. How would you rate the level of difficulty of virtual patients in this course?

Very difficult	8	1.7%
Difficult	93	19.4%
Neither easy nor difficult	301	62.8%
Easy	54	11.3%

Very easy	4	0.8%
[Blank]	19	4.0%
